# Comparative transcriptomics among floral organs of the basal eudicot *Eschscholzia californica *as reference for floral evolutionary developmental studies

**DOI:** 10.1186/gb-2010-11-10-r101

**Published:** 2010-10-15

**Authors:** Laura M Zahn, Xuan Ma, Naomi S Altman, Qing Zhang, P Kerr Wall, Donglan Tian, Cynthia J Gibas, Raad Gharaibeh, James H Leebens-Mack, Claude W dePamphilis, Hong Ma

**Affiliations:** 1 Department of Biology, The Pennsylvania State University, University Park, PA 16802, USA; 2 The Huck Institutes of the Life Sciences, The Pennsylvania State University, University Park, PA 16802, USA; 3 The Intercollege Graduate Program in Cell and Developmental Biology, The Pennsylvania State University, University Park, PA 16802, USA; 4 Department of Statistics, The Pennsylvania State University, University Park, PA 16802, USA; 5 Department of Bioinformatics and Genomics, The University of North Carolina at Charlotte, 9201 University City Boulevard, Charlotte, NC 28223, USA; 6 State Key Laboratory of Genetic Engineering and School of Life Sciences, Fudan University, 220 Handan Road, Shanghai 200433, China; 7 Institutes of Biomedical Sciences, Fudan University, 138 Yixueyuan Road, Shanghai 200032, China; 8 Current address: American Association for the Advancement of Science, 1200 New York Avenue NW, Washington DC 20005, USA; 9 Current address: 2367 Setter Run Lane, State College, PA 16802, USA; 10 Current address: BASF Plant Science, 26 Davis Drive, Research Triangle Park, NC 27709, USA; 11 Current address: Department of Entomology, The Pennsylvania State University, University Park, PA 16802, USA; 12 Current address: Department of Plant Biology, University of Georgia, 120 Carlton Street, Athens, GA 30602, USA

## Abstract

**Background:**

Molecular genetic studies of floral development have concentrated on several core eudicots and grasses (monocots), which have canalized floral forms. Basal eudicots possess a wider range of floral morphologies than the core eudicots and grasses and can serve as an evolutionary link between core eudicots and monocots, and provide a reference for studies of other basal angiosperms. Recent advances in genomics have enabled researchers to profile gene activities during floral development, primarily in the eudicot *Arabidopsis thaliana *and the monocots rice and maize. However, our understanding of floral developmental processes among the basal eudicots remains limited.

**Results:**

Using a recently generated expressed sequence tag (EST) set, we have designed an oligonucleotide microarray for the basal eudicot *Eschscholzia californica *(California poppy). We performed microarray experiments with an interwoven-loop design in order to characterize the *E. californica *floral transcriptome and to identify differentially expressed genes in flower buds with pre-meiotic and meiotic cells, four floral organs at pre-anthesis stages (sepals, petals, stamens and carpels), developing fruits, and leaves.

**Conclusions:**

Our results provide a foundation for comparative gene expression studies between eudicots and basal angiosperms. We identified whorl-specific gene expression patterns in *E. californica *and examined the floral expression of several gene families. Interestingly, most *E. californica *homologs of *Arabidopsis *genes important for flower development, except for genes encoding MADS-box transcription factors, show different expression patterns between the two species. Our comparative transcriptomics study highlights the unique evolutionary position of *E. californica *compared with basal angiosperms and core eudicots.

## Background

**Figure 1:**
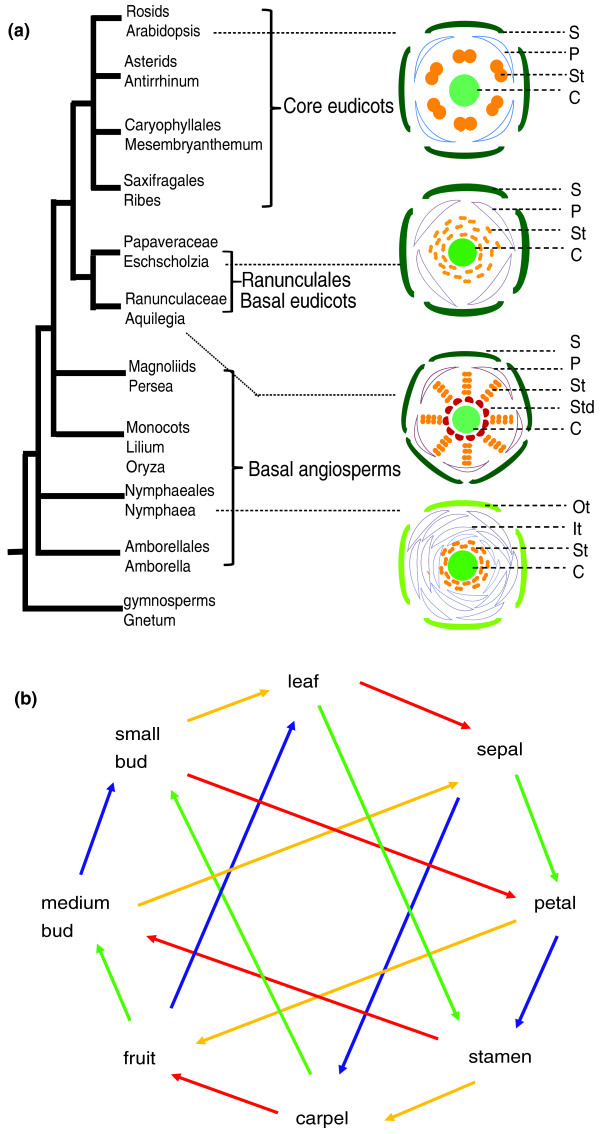
**An angiosperm phylogram with illustrations of flower structures and the loop design of the *E. californica *microarray experiments**. **(a) **A phylogram of angiosperms with flower architectures for several representative species. C, carpel; It, inner tepals; Ot, outer tepals; P, petal; S, sepal; St, stamen; Std, staminodia. **(b) **We sampled from eight different tissues, including leaves, small floral buds, medium floral buds, four floral organs (sepals, petals, stamens, and pistils) at anthesis, and young fruits (four replicates for each tissue, 32 in total). Each line connects samples from two tissues in one microarray hybridization reaction, and four different colors represent four replicates of each tissue. The points of the arrows point to the samples labeled with Cy5 dyes while the bases of the arrows point to the samples labeled with Cy3 dyes.

The eudicots are believed to have originated approximately 130 million years ago [1]. They include about 70% of all flowering plant species and consist of core eudicots [2-4], which include the groups containing *Arabidopsis thaliana *and *Antirrhinum majus*, and species that branched earlier from these groups and are at basal positions within the eudicot clade. The earliest branching lineage of the eudicots, the Ranunculales, contains the Papaveraceae (poppy) family, of which *Eschscholzia californica *(California poppy) is a member [3]. The core eudicots commonly have stable (that is, canalized) flower architecture (Figure 1a); by contrast, the basal eudicots exhibit a wider range of floral patterns [5] (see examples in Figure 1a). Comparing the morphology and the underlying mechanisms of flower development between the core and basal eudicots may help us better understand the evolution of flower structures and development.

Molecular genetic studies in *Arabidopsis*, *Antirrhinum *and other core eudicots have uncovered the functions of many genes involved in regulating flowering time and floral organ identity and development [6-8]. In particular, it is known that several MADS-box genes are required to control flowering time and floral organ identities, as well as anther, ovule and fruit development. These include the well-known ABC genes *APETALA1 *(A function), *APETALA3 *and *PISTILLATA *(B function), and *AGAMOUS *(C function) from *Arabidopsis*, and their respective functional homologs from *Antirrhinum *(*SQUAMOSA*, *DEFICIENS*, *GLOBOSA*, and *PLENA*) [9-11]. Comparative studies of core eudicots suggest that homologs of B- and C-function genes have relatively conserved functions, although some divergences have also been observed. Putative orthologs of these MADS-box genes may have diverged expression patterns in different species and the expression difference between recent duplicates is often associated with subfunctionalization [10,11]. In addition, several MADS-box genes have been found to be important for floral organ identities in the monocots [12-15]. However, both the long evolutionary distance and the highly diverged flower architectures between monocots and core eudicots have made it difficult to study the evolution of floral gene function.

The investigation of floral gene function in the basal eudicots serves to bridge the gap between core eudicots and monocots. Molecular and expression studies of floral genes have been reported for some basal eudicots, providing informative initial knowledge on the conservation and divergence of floral gene activities among eudicots [16-18]. Molecular evolutionary studies of several MADS-box subfamilies, complemented by expression analyses, support that some of the MADS-box genes have maintained conserved functions throughout angiosperm evolution [10,19-22]. For example, expression studies of floral MADS-box genes in *E. californica *demonstrated that genes in the *AGAMOUS*, *GLOBOSA *and *SEPALLATA *subfamilies are highly conserved between basal and core eudicots [10,11,20]. Additionally, in other ranunculids, expression divergences have also been observed between recently duplicated MADS-box genes [10,11].

High-throughput technologies, including microarrays, can be used to analyze transcriptomes of individual floral organs at specific developmental stages. Transcriptome studies have been performed extensively for *Arabidopsis *and, to a lesser extent, several other highly derived core eudicots [18,23-28]. Among basal eudicots, such studies have only been carried out recently in the basal eudicot *Aquilegia*, which represents a different ranunculid lineage than *E. californica *[29]. *E. californica *is a potential model organism because it has a relatively small plant size, many seeds per fruit and a short generation time, which facilitate genetic studies; because it does not have determinate flowering and produces multiple flowers over its lifespan, providing easy access to floral materials [30]; because it has a relatively small genome; and because it both has an efficient system for virally induced gene silencing and is transformable [20,31-34]. Previous gene expression studies in *E. californica *showed that there is very good correlation between regions of gene expression and domains of gene function [18,33,35,36]. An *E. californica *EST collection of over 6,000 unigenes was constructed from a pre-meiotic floral cDNA library [20], which provides gene sequence information for microarray analysis of *E. californica *leaf and floral transcriptomes. A transcriptome-level analysis facilitates our understanding of floral development in basal eudicots and sheds light on potential floral regulatory genes in *E. californica*.

In this study, we used microarray technology to investigate transcriptomes in *E. californica *and to identify differentially expressed genes in developing leaves and floral buds at pre-meiotic (small buds) and meiotic (medium buds) stages. Additionally, we examined the transcriptomes of developing fruits and four types of floral organs (sepals, petals, stamens, and carpels) at the pre-anthesis stage. We identified genes that are significantly differentially expressed in different floral organs or at different floral stages, in comparison with developing fruit and leaf tissues. We also analyzed the expression of genes in several regulatory gene families, some of which contain homologs of known floral genes from other organisms. Finally, we compared our results with similar studies in *Arabidopsis *and recent studies [29,37] in *Aquilegia *and *Persea *(avocado), a basal angiosperm related to magnolia, to assess conservation and divergence in gene expression and discuss their implications for evolution of floral development in the eudicots.

## Results and discussion

### Construction and use of a microarray chip for *E. californica*

To investigate the leaf and reproductive transcriptomes of *E. californica*, we generated a custom Agilent microarray chip with features for 6,446 unigenes from the *E. californica *EST collection [20] (see Materials and methods for additional information). The oligonucleotide sequences for the probes were selected using available sequence information from *E. californica *ESTs, as well as other public sequence information, avoiding non-specific hybridization as much as possible. Additional criteria were used to consider potential secondary structure and hybridization temperature (see Materials and methods).

A primary objective was to obtain expression profiles with the power to detect differential expression between vegetative (leaves) and reproductive organs, between different floral stages, and between different floral organs. Therefore, we sampled the *E. californica *plants for the following eight representative organs and stages (for convenience, referred to generally as tissues hereafter): leaves, early floral buds, medium floral buds, four floral organs (sepals, petals, stamens, and carpels) at pre-anthesis, and young fruits. Four sets of plants were sampled at the same time daily (8:30 to 10:30 am) to minimize variation due to circadian rhythms, yielding four biological replicates. RNAs from these 32 samples were used to generate cDNAs and labeled with Cy3 and Cy5 dyes for two-channel microarray experiments. Finally, we used an interwoven loop design (Figure 1b) to maximize the comparative statistical power using a limited number of hybridizations [38].

In an interwoven loop design, differences in gene expression can be estimated for all pairs of tissues with a relatively small number of hybridizations [39]. Each of the eight tissues was directly compared on the same slide with one of four other tissues, with one biological replicate for each comparison, resulting in a total of 16 hybridizations. The comparison of the two tissues on the same arrays allowed more precise results than those compared indirectly via other tissues. The specific pairings on the same array were chosen to optimize precision of comparisons for biologically important comparisons, while keeping the precision of different comparisons as similar as possible. Because our EST library was constructed with floral bud mRNAs, we compared developing floral buds at different stages with each of the four floral organs, and compared each of these tissues with leaves, the only vegetative organ in this study, and developing fruits. The comparison between small buds and leaves was aimed at identifying differentially expressed genes at early reproductive stages. We hypothesized that the sepal should be the most leaf-like tissue among all floral organs; whereas previous studies [24] suggest that the stamens might have the most complex transcriptome among the four major floral organs [26]. In this study, the fruit tissue represents the only post-anthesis tissue. We also considered the ABC model, which posits that sepals and petals both require A-function genes, petals and stamens both need B-function genes, and stamens and carpels both depend on C-function genes. In addition, carpels and fruits were developmentally related tissues, with small and medium buds representing two consecutive stages in floral development.

**Figure 2:**
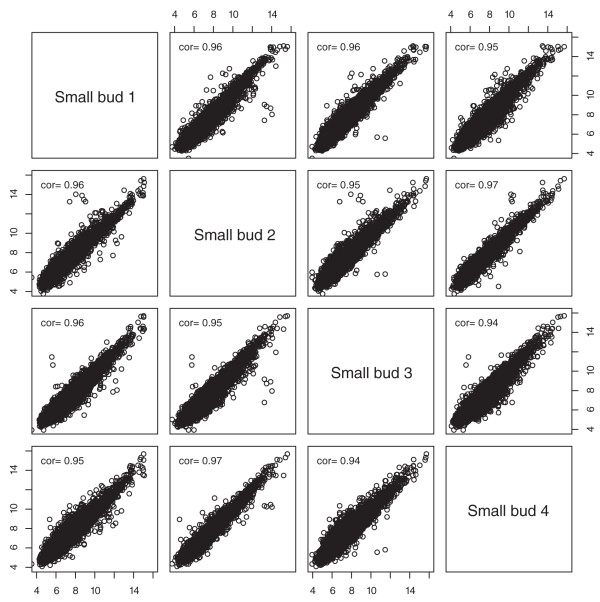
**Correlation coefficients between signal intensities from four biological replicates of the small floral buds**. Pearson's correlation coefficients were between 0.94 and 0.97 between any pair of the four biological replicates, indicating that the results were highly reproducible.

After microarray hybridizations, we tested the quality of the microarray experiments. We assessed the reproducibility of the microarray hybridizations by determining the Pearson's correlation coefficients between the biological replicates for each of the eight tissues (see Figure 2 for an example; the plots for the remaining seven tissues can be found in Figure S1 in Additional file 1). As shown in Figure 2, the Pearson's correlation coefficients between any pair of the four biological replicates of small buds, one of the most complex tissues in this study, ranged from 0.94 to 0.97. The high correlation values indicate that our results were highly reproducible.

**Figure 3:**
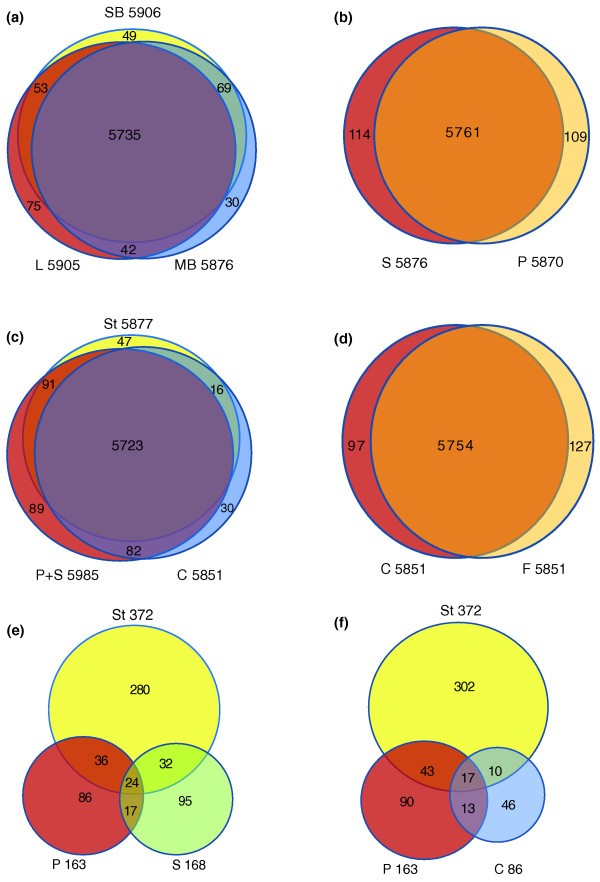
**Venn diagrams of genes expressed in reproductive tissues**. **(a**-**d) **Genes expressed in different tissues and their intersections. **(e-f)** Genes significantly preferentially expressed compared with leaf with more than two-fold differences and their intersections. C, carpel; F, fruit; L, leaf; MB, medium bud; P, petal; S, sepal; SB, small bud; St, stamen.

In addition, we examined signal intensities. Because the EST library used for the probe design was constructed from mRNAs of flower buds, we assumed that expression of most genes should be detected in our microarray experiments from mostly flower-related tissues. The value of 5.41 for log2 of hybridization intensity (10% quantile of all genes on the chip) was selected as a cutoff to identify 'present' signal (Table 1; for alternative cutoffs, see Additional file 2 for gene numbers with 5% or 15% quantiles) similar to previous microarray experiments in *Arabidopsis *[28]. For the 10% quantile, we identified the number of genes detected in leaves (5,905), small buds (5,906), medium buds (5,876), sepals (5,876), petals (5,870), stamens (5,877), carpels (5,851) and fruits (5,881). These results were not surprising because the unigenes were derived from EST data, which tend to favor genes that are expressed at relatively high levels. Therefore, our microarray chip and hybridization experiments were able to detect the expression of several thousand genes in eight major tissues of *E. californica*. Of the genes examined, the majority of genes present in leaf were also observed in small buds and medium buds (Figure 3a). In addition, most genes expressed in sepal were also expressed in petal (Figure 3b), suggesting similar gene expression levels between these two tissues. There was significant overlap of genes expressed in petal and/or sepal with genes expressed in carpel and stamen (Figure 3c). Similarly, there was considerable overlap of expressed genes between the carpel and fruit (Figure 3d); this is not surprising since fruit is derived from the ovary containing large carpel tissues. Using the same cutoff for detection of expression, 5,554 genes were expressed in all 8 tissues (Table S1 in Additional file 2). We then examined Gene Ontology (GO) categorization of all 5,554 genes and found that the 'unknown' genes (homolog of genes annotated as unknown in *Arabidopsis*) were under-represented while some specific functional categories were slightly over-represented, including transferase and protein binding group (Additional file 3 and Figure S2 in Additional file 1). The observation that most of the genes in this study were expressed in all tissues might be because our EST collection represented relatively abundant genes, including most house-keeping genes. This might also explain why the 'unknown' category was under-represented because widely expressed genes tend to have known annotations.

**Table 1:** California poppy genes preferentially expressed in pre-meiotic and meiotic stage buds and in fruit

Gene	BestATHit	L	SB	MB	S	P	ST	C	F	Annotation
Preferentially expressed in pre-meiotic buds										
89282	AT2G31210.1	5.3	9.0	7.1	5.6	5.3	5.3	5.3	5.1	bHLH
83967	AT5G16920.1	7.1	9.9	8.5	7.3	7.0	6.9	6.9	6.9	
84082	AT1G68540.1	6.8	10.2	8.9	6.8	6.9	6.7	6.5	6.2	Oxidoreductase
87393	AT1G44970.1	5.1	7.9	5.9	5.1	5.0	5.0	5.6	5.2	Peroxidase
86946	AT4G33870.1	7.8	9.5	8.1	8.0	7.8	7.9	7.8	7.8	Peroxidase
86850	AT3G28470.1	6.2	7.5	6.4	6.1	6.1	6.1	6.1	6.0	ATMYB35
85123	AT5G09970.1	5.9	9.5	7.6	5.4	5.4	5.1	6.5	7.3	CYP78A7
Preferentially expressed in meiotic buds										
84975	AT5G35630.2	6.9	6.7	8.5	6.8	6.6	6.7	6.6	6.9	GS2
85233	AT1G11910.1	5.6	7.4	10.2	9.1	6.1	8.5	6.1	8.4	Aspartyl protease
86094	AT1G54220.1	6.8	7.8	9.9	7.5	7.3	8.6	7.0	7.2	Dihydrolipoamide S-acetyltransferase
88004	AT4G16260.1	5.7	7.5	9.7	6.0	5.9	6.1	5.4	5.8	Hydrolase
88092	AT4G12910.1	9.1	9.3	10.9	8.9	8.5	8.4	9.0	9.4	scpl20
88096	AT3G11450.1	7.8	8.2	9.9	7.8	7.8	8.2	7.9	7.9	Cell division protein-related
88675	AT4G35160.1	6.3	6.6	7.9	6.6	6.3	6.2	6.1	6.2	O-methyltransferase
89901	AT5G03880.1	7.6	7.6	8.7	7.7	7.4	7.6	7.3	7.5	Electron carrier
Preferentially expressed in fruits										
83998		6.4	5.8	5.7	6.3	6.5	5.8	6.2	8.5	
84097	AT5G54160.1	9.4	9.1	10.0	9.1	8.6	8.1	9.0	11.1	ATOMT1
86118	AT5G62200.1	7.6	7.0	7.4	7.6	7.6	7.7	7.3	9.3	Embryo-specific protein
86486	AT1G07080.1	6.5	6.6	6.9	6.8	6.3	6.8	6.6	10.1	GILT
87027		5.8	5.5	5.5	5.7	5.6	6.0	5.8	7.3	
87195	AT5G12380.1	6.6	6.2	6.5	6.7	6.4	6.5	7.2	9.6	Annexin
87830	AT5G08260.1	6.0	5.9	6.2	6.0	6.1	5.9	6.1	7.4	scpl35
88106	AT1G20030.2	6.6	6.3	6.8	7.3	5.9	6.4	6.5	9.0	Pathogenesis-related thaumatin
89333		8.8	6.5	8.0	8.4	5.9	7.6	7.1	10.5	

The first column is the gene number for genes represented by poppy ESTs. The second column is the closest *Arabidopsis *homolog of each poppy gene. All expression values are log2 ratio. C, carpel; F, fruit; L, leaf; MB, medium bud; P, petal; S, sepal; SB, small bud; ST, stamen. Annotations are from TAIR version 9.

To verify our microarray results, real-time reverse-transcription PCR (RT-PCR) was performed using RNAs from the same eight tissues as those in microarray experiments. Nine representative genes were examined relative to our reference gene (Figure S3 in Additional file 1), including three MADS-box genes, *EScaAGL2 *(87251), *EScaAGL6 *(86583), and *EScaDEF1 *(83744) [10]. The other genes were homologs of a transcription factor MYB35 (86850), a gamma-tip protein (84392), a putative ferrodoxin (85140), a transducin family/WD-40 repeat family protein (84618), and homologs (86386 and 88941) of two *Arabidopsis *genes encoding different 'expressed proteins' without a known function. The real time RT-PCR results indicate that the gene expression patterns were generally supportive of the microarray results, and were also consistent with previous RNA *in situ *hybridization experiments [10,11,40,41].

### An overview of differential expression profiling of floral development

Although the *E. californica *ESTs were obtained from a cDNA library that was constructed with mRNAs from multiple stages of floral development [20], many of the corresponding genes were also expressed in leaves, different stages and various organs of the flower, as well as fruits. To determine additional transcriptome characteristics, we investigated whether specific genes were expressed similarly or differentially in the tissues tested. Of the 6,446 unigenes examined, most genes (4,513 of 6,446) were not significantly differentially expressed with more than a two-fold change between any two of the eight tissues (with *P*-value < 0.05).

**Figure 4:**
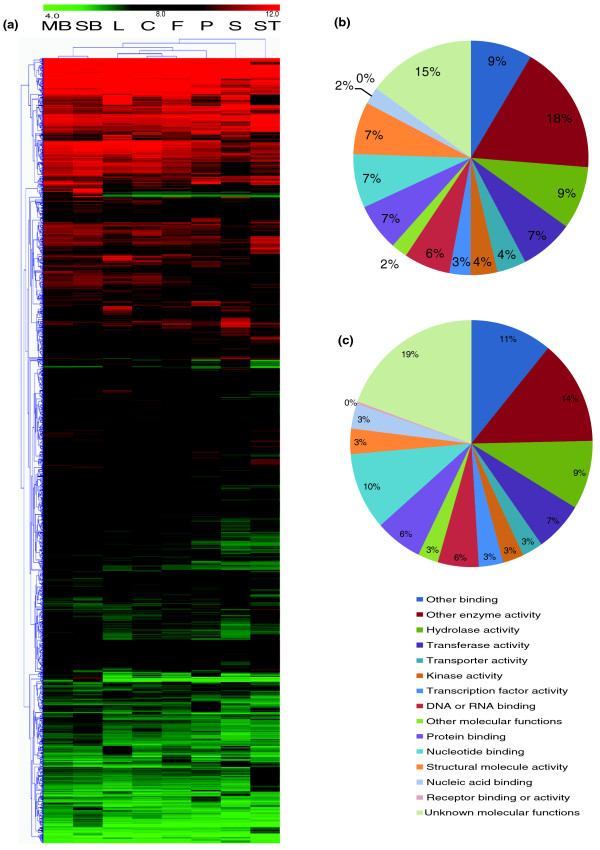
**Heat maps and GO annotation pie chart of genes differentially expressed between any two tissues**. **(a) **Heat map for the mRNA profiles of 1,921 genes differentially expressed between any two tissues. Red color represents high expression while green color represents low expression. HCL clustering was performed on transcript ratios of all tissues across tissues and genes. Two major clusters had been identified as C1 and C2. C, carpel; F, fruit; L, leaf; MB, medium bud; P, petal; S, sepal; SB, small bud; ST, stamen. **(b) **GO categorization of all *Arabidopsis *homologs of poppy genes included in our chip as control. **(c) **GO categorization of all *Arabidopsis *homologs of poppy genes that were statistically significantly differentially expressed.

Nevertheless, 1,933 genes were found to be differentially expressed between at least two tissues (Table S2 in Additional file 4); however, most of these 1,933 genes showed similar expression levels in the other tissues (Figure 4a). Not surprisingly, carpel and fruit, as well as small and medium buds, showed the most similar expression patterns at sequential development stages. Leaf, the only vegetative organ in our study, had similar expression patterns to those of the green organs (carpel and fruit), which may be due to shared high expression of photosynthesis-related genes (see below). Interestingly, stamen had the most different expression profile, suggesting a distinct developmental process relative to the other floral organs.

To obtain additional insights into functions of those differentially expressed genes, we examined the GO categorization for the most similar *Arabidopsis *homologs of each poppy gene using functions within The *Arabidopsis *Information Resource (TAIR) website [42] (Additional file 3). Genes encoding proteins categorized as 'other enzyme activity' (chi-square test with *P*-value < 0.01) and 'structural molecule' (*P*-value < 0.001) were enriched among those genes differentially expressed between at least two tissues (Figure 4c) relative to the control group of all genes on the microarray chip (Figure 4b). These results suggested that variation in the expression of metabolic genes across those tissues might be responsible, in part, for their morphological and/or physiological differences in *E. californica*.

### Similar expression pattern of vegetative preferential genes in *E. californica *and in *Arabidopsis*

**Figure 5:**
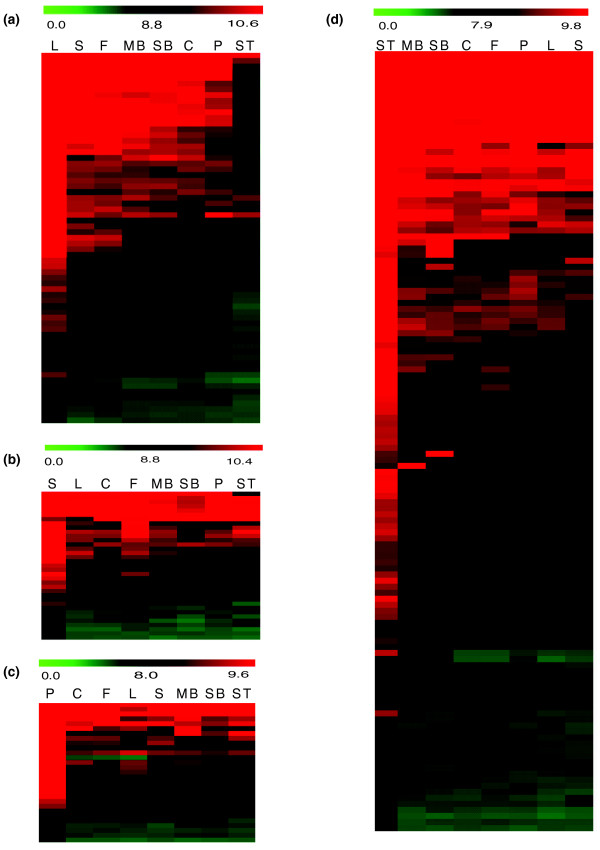
**Heat maps of genes preferentially expressed in different tissues**. Red color represents high expression while green color represents low expression. **(a-c) **Heat map of genes preferentially expressed in leaf compared with all the other tissues (a), sepal compared with all the other tissues (b), and petal compared with all the other tissues (c). **(d) **stamen compared with all the other tissues. C, carpel; F, fruit; L, leaf; MB, medium bud; P, petal; S, sepal; SB, small bud; ST, stamen.

To identify genes with greater expression in either vegetative or reproductive tissues, we performed pairwise comparisons among all tissues as well as groups of floral organs and/or stages. Only one gene, 90036 (with no significant BLASTX hits to *Arabidopsis *predicted proteome, nor the NCBI NR database), was significantly twofold greater in all reproductive tissues and through all stages, including fruit, compared to leaf tissue. However, 65 genes were expressed significantly higher in leaves compared to all floral tissues and stages (Table S2 in Additional file 4). To obtain overall expression patterns of vegetative genes, we constructed a heat-map (Figure 5a) resulting in two main clusters. In the first cluster, most genes that were highly expressed in leaves were also highly expressed in floral tissues except stamens. In the second cluster, most genes were highly expressed in leaves but not in the other tissues.

To compare gene expression pattern of leaf-preferential genes in *E. californica *and their homologs in *Arabidopsis*, we used BLAST to search the *E. californica *EST sequences against the *Arabidopsis *genome. Our BLAST results (with 10E^-10 ^as cutoff) indicate that 58 out of the 65 leaf-preferential genes have identifiable homologs in *Arabidopsis*. On the basis of previous microarray data, of these 58 genes all but one (*RBCS1A*) of their *Arabidopsis *homologs were also preferentially expressed in leaves (Table S4 in Additional file 5) [43]. According to TAIR9 annotation, most of these genes encode proteins that are localized in the chloroplast. GO categorization on the basis of gene function (methods) indicate that most of these genes are likely to be involved in photosynthesis, encoding homologs of protochlorophyllide reductases, photosystem I reaction center subunits and oxygen-evolving enhancer proteins.

### Comparing transcriptome profiles at crucial stages of floral development in *E. californica *and in *Arabidopsis*

To identify developmental stage-specific genes in *E. californica *flowers, we examined the expression patterns of genes in the pre-meiotic (small buds), meiotic (medium buds) and pre-anthesis stages (four floral organs: sepals, petals, stamens and carpels). Pre-meiotic buds (small buds < 5 mm) had 49 differentially expressed genes in comparison with any other tissues examined (*P*-value < 0.05 and two-fold cutoff; Table S2 in Additional file 4). Among these genes, 30 had identifiable *Arabidopsis *homologs, 24 of which have expression data available (Table S4 in Additional file 5). Unlike leaf-preferential genes, only 7 of these 24 genes showed expression peaks in early *Arabidopsis *flower buds while the rest were predominately expressed in specific floral organs at higher levels than in leaves. The proteins encoded by these seven genes include two transcription factors, one oxidoreductase, two peroxidases, one electron carrier and one gene of unknown function (Table 1, genes and annotations with peak expression in small floral buds; information obtained from Markus Schmid's results [43]. The *Arabidopsis *homologs for two transcription factors, *MYB35*, which regulates anther cell layer formation at early stages, and a basic helix-loop-helix (bHLH) gene that has not been fully studied [44,45], were also preferentially expressed in anthers (X Ma and B Feng, unpublished data). However, the corresponding *E. californica *genes were expressed at low levels in the pre-anthesis stamens, possibly because either these genes are not highly expressed in *E. californica *stamens or our stamen expression data from pre-anthesis stamens were too late relative to the stages of highest expression in *Arabidopsis*, which may be during earlier anther developmental stages.

In medium buds (which span the meiotic stage), we found eight genes that were expressed twofold significantly higher and none that were significantly down-regulated compared with any of the other tissues examined (Table 1). All of these genes have homologs in *Arabidopsis *and most encode proteins that may have enzymatic activities (Table 1). However, none of the *Arabidopsis *homologs of these genes show expression peaks in the equivalent stages to our medium buds in *Arabidopsis *[43] (Table 1; Table S4 in Additional file 5). Interestingly, the homolog of *E. californica *gene 88096 in *Arabidopsis *(AT3G11450) encodes a DnaJ heat shock protein proposed to be involved in either mitosis or meiosis. The expression pattern of these homologs differs in that it is highly expressed in both vegetative and reproductive tissues in *Arabidopsis*. It is possible that the gene function might have diverged after the separation of basal eudicots from core eudicots.

In fruits, nine genes were expressed significantly twofold higher than the other tissues in *E. californica *(Table 1). None of their homologs showed an expression peak in the *Arabidopsis *fruit. Among the genes of particular interest, the *Arabidopsis *homolog of 86118 (At5g62200, MMI9) plays an important role in embryo development [46], and its high expression in the fruits suggests that its *E. californica *homolog might have a similar function.

### Identification of putative genes under control of certain genes in the ABC model

According to the ABC model, A-function genes are transcription factors that are required to properly specify the sepal (alone) and petal (along with B-function genes) identities, with B-function genes specifying the stamen (along with C-function genes), and C function specifying the carpel. Thus, genes expressed in sepals and petals (regions encompassing the A domain) are called A-domain genes, genes expressed in petals and stamens are called B-domain genes, and genes expressed in stamens and carpels are called C-domain genes. Although the homologs of *Arabidopsis *A-function genes (such as *AP1 *and *AP2*) might not have conserved functions in other eudicots [45-47], because of the distinct sepals and petals in *E. californica*, we tried to identify putative A-function genes on the basis of regulatory genes expressed in the A domain, hypothesizing that they may function in specifying the sepal and petal identities in *E. californica*.

**Figure 6:**
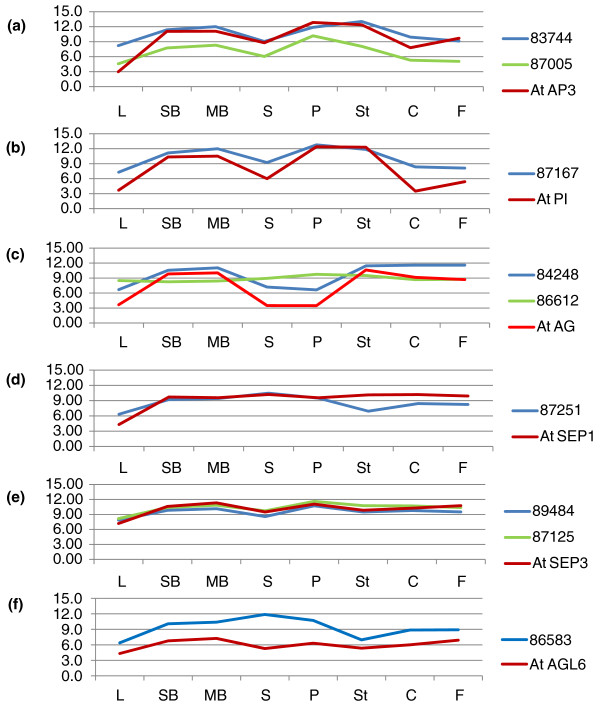
**The expression levels of MADS transcription factor families**. **(a-f)** Expression of *E. californica *homologs of B function genes *AP3 *(a) and *PI *(b), C function gene *AG *(c), E function genes, *SEP1 *(d) and *SEP3 *(e), and *AGL6 *(f) in eight tissues compared with their counterparts in *Arabidopsis*. All the expression values are log2 ratio. The y-axis is the log2 ratio of gene expression levels. C, carpel; F, fruit; L, leaf; MB, medium bud; P, petal; S, sepal; SB, small bud; St, stamen.

From our hypothesis that A-domain genes should be more highly expressed in sepals, and possibly in petals, than in the other floral organs, we compared them with three tissues: leaf, stamen and carpel collected approximately 1 day pre-anthesis. We found significantly greater expression of 64 genes in sepals over each of the above 3 tissues and 49 genes in petals over each of the 3 tissues, respectively (Table S5 in Additional file 6). When compared with all 7 other tissues, 34 genes in sepals and 29 genes in petals were significantly preferentially expressed (Table S2 in Additional file 4). Whereas genes highly expressed in sepals or petals tended to be expressed in all tissues at moderately high levels (Figure 5b,c), genes with lower expression in sepals and/or petals were scarcely expressed in other tissues. On the basis of comparisons of petals and sepals with leaves, stamens and carpels, only five genes were expressed twofold greater in tissues controlled by A-function genes (Table 2). Interestingly, two of these genes are members of the MADS-box family. However, the expression of their closest *Arabidopsis *homologs, *AGL2*/*SEP1 *and *AGL6*, is not sepal-, petal- or even floral-specific (Figure 6d,f). *SEP1 *is an E-function gene [47,48], and is involved in the development of all floral organs in *Arabidopsis*. A homolog of *SEP1 *in soybean (*GmSEP1*) is expressed in reproductive development, especially in petals and seed coats [49]. *AGL6 *and its homologs have been shown to function in flower development not only in eudicots, like *Arabidopsis *and *Petunia*, but also in orchid, rice, and other monocots. In the grasses, *AGL6 *has high expression in paleas, lodicules, carpels and ovule integuments, as well as the receptacle [50-54]. We hypothesize that other *MADS *genes, possibly *SEP *homologs, may serve as A-function genes in *E. californica *instead of *AP1 *and *AP2 *in *Arabidopsis*, in part because the *AP1 *subfamily is closely related to the *AGL6 *and *SEP *subfamilies [55].

**Table 2:** Expression levels of putative ABC genes in poppy

Gene	BestATHit	L	SB	MB	S	P	ST	C	F	Annotation
A-function genes										
84392	AT2G36830.1	14.4	13.9	14.3	16.2	16.3	15.1	14.5	14.4	GAMMA-TIP
86583	AT2G45650.1	6.4	10.1	10.4	11.9	10.7	7.0	8.9	8.9	AGL6
87043	AT3G05490.1	8.9	9.1	9.8	10.6	10.9	9.6	9.2	9.3	RALFL22
87251	AT5G15800.1	6.3	9.2	9.4	10.5	9.5	6.9	8.4	8.3	SEP1, AGL2
85671		7.3	7.0	6.9	10.5	11.1	8.4	7.3	7.7	
B-function genes										
83744	AT3G54340.1	8.2	11.4	12	9.1	11.9	13.1	9.9	9.1	AP3
83763	AT1G69500.1	5.2	5.7	6.1	5.9	7.7	7.3	6.1	5.7	Electron carrier
83991	AT5G19770.1	10.0	10.1	10.3	9.0	11	11.2	9.8	10.1	TUA3
84789	AT5G64250.2	11.9	11.4	13.2	13.8	15.6	15.0	13.9	13.2	2-Nitropropane dioxygenase
85140	AT2G27510.1	9.2	11.1	12.2	10.8	13.8	11.9	10.5	10.0	Ferredoxin 3
85166	AT5G62690.1	9.2	10.0	10.2	8.3	10.4	10.9	9.3	9.8	TUB2
85610	AT4G36250.1	5.4	6.5	8.5	6.4	7.8	7.6	6.1	5.5	Aldehyde dehydrogenase 3F1
87005	AT3G54340.1	4.6	7.8	8.3	6.0	10.2	8.1	5.3	5.1	AP3
87035	AT3G58120.1	5.6	5.5	5.7	5.5	7.9	8.0	5.1	5.2	ATBZIP61
87167	AT5G20240.1	7.3	11.2	12.0	9.2	12.8	11.9	8.4	8.1	PI
87294	AT5G03690.2	8.0	9.3	10.0	7.8	9.8	10.0	8.7	9.2	Fructose-bisphosphate aldolase
89750	AT4G37990.1	8.2	8.8	9.6	8.5	11.4	10.2	7.7	7.9	Mannitol dehydrogenase
89805	AT5G66310.1	5.9	6.5	6.8	5.3	7.2	7.8	6.1	6.4	Kinesin motor
C-function genes										
84248	AT4G18960.1	6.7	10.6	11.1	7.2	6.6	11.5	11.6	11.6	AG
84252	AT4G26220.1	7.3	10.9	10.9	7.0	7.2	10.9	10.1	6.6	Caffeoyl-CoA 3-O-methyltransferase
84340	AT3G44260.1	7.9	8.4	8.3	7.8	8	9.9	9.2	8.6	CCR4-NOT transcription complex protein
84512	AT1G11910.1	7.2	9.5	10.1	7.0	7.2	8.8	9.1	9.1	Aspartyl protease
84691	AT2G44480.1	9.1	12.4	12.8	8.7	8.6	12.6	12	12.9	BETA GLUCOSIDASE 17
89115	AT3G20240.1	6.4	7.4	7.3	6.3	6.2	8.2	7.6	7.0	Mitochondrial substrate carrier
89980	AT1G35720.1	7.1	8.8	9.5	7.2	7.9	10.1	9.2	8.5	ANNEXIN ARABIDOPSIS 1

The first column is the gene number for genes represented by poppy ESTs. The second column is the closest *Arabidopsis *homolog of each poppy gene. All expression values are log2 ratio. C, carpel; F, fruit; L, leaf; MB, medium bud; P, petal; S, sepal; SB, small bud; ST, stamen. Annotations are from TAIR version 9.

B-function genes, such as the *Arabidopsis APETALA3 *and *PISTILLATA *genes, are required for the identities of petals and stamens [9,11,56]. In monocots like tulip, homologs of *AP3 *and *PI *are expressed in the tepals (petal-like organs found in the outer two whorls). We searched for putative B-domain genes on the basis of their expression patterns in *E. californica *and found that 60 genes in petals and 180 genes in stamens were expressed significantly higher in these organs than in sepals, carpels and leaves (Table S5 in Additional file 6). And 94 genes were expressed twofold significantly greater in stamens than all the other organs (Table S2 in Additional file 4). The large number of genes with stamen-preferential expression patterns suggests that the development of stamen requires more specialized genes. Alternatively, the larger number of stamen-preferential genes identified here may be explained by the fact that stamens comprise much of the biomass of developing *E. californica *buds, relative to other developing floral organs (Figure 5d).

We combined the expression data from petals and stamens to represent the B-domain group and compared their expression levels with those of leaves, sepals, carpels and fruits (Table 2), identifying 13 genes as preferentially expressed in the B-domain organs. A homolog of *PI *(87167) and two homologs of *AP3 *(83744 and 87005) were identified in this group [11] (Table S5 in Additional file 6). Since *PI *and *AP3 *are B-function genes in *Arabidopsis *and other species, such as lily [57-59], it is possible that their homologs in *E. californica *function in a similar manner. It should also be noted that *in situ *analysis showed that the *AP3 *homologs are also expressed in ovules in *E. californica *[11], suggesting that they may have roles outside of B-function.

Of the genes preferentially expressed in the B-domain, one is a homolog of the At*bZIP61 *gene, which encodes a putative transcription factor and is expressed in *Arabidopsis *flowers, with especially high expression in petals. It is not known whether At*bZIP61 *regulates floral development in *Arabidopsis*. However, on the basis of its expression pattern and that of its homolog in *E. californica*, we speculate that it functions to regulate petal development and is downstream of the B-function genes.

In *Arabidopsis*, C function is controlled by *AGAMOUS*, which specifies stamens and carpels. When compared with leaves, sepals and petals, 26 genes were preferentially expressed in carpels (compared to 168 genes in stamens; Table S5 in Additional file 6). We searched for C-domain genes and found that seven genes (Table S5 in Additional file 6) were expressed twofold significantly greater in stamens and carpels than in leaves, sepals and petals. Among them was a homolog of the *Arabidopsis *C-function gene *AG *[59]. Since both monocots (rice) and other eudicots have *AG *homologs functioning in stamen and carpel development, we hypothesize that the *AG *homolog in *E. californica *has similar functions [10,60,61]. It has been proposed that D-domain genes are required for ovule development, but only one *E. californica *gene (88769) was expressed in carpels twofold significantly higher over all other tissues. This EST did not have an identifiable *Arabidopsis *homolog.

To uncover additional candidates of A-, B- or C-domain genes, we used less stringent criteria and selected genes with expression levels at least twofold higher in each pre-anthesis reproductive tissue than in leaves (with false discovery rate (FDR) < 0.05; Figure 3e,f; Table S6 in Additional file 7). We found that most of these genes were expressed in a whorl-specific manner and only a small numbers of genes were co-upregulated in sepals and petals, in petals and stamens, or in stamens and carpels. Furthermore, the overlap of A/B-domain and that of B/C-domain genes were even smaller (Figure 3e,f). Unlike studies in *Persea *and *Aquilegia*, whose floral transcriptomes were interpreted as support for a 'fading borders' model of floral organ identity [29,62], the *E. californica *floral transcriptomes were rather distinctive, providing a molecular explanation for the morphologically different sepals and petals. Therefore, *E. californica *might have adopted an ABC model with relatively sharp borders, similar to those found in core eudicots. Because *E. californica *is basal to *Aquilegia *within the Ranunculales, as determined by phylogenetic analyses [37], it may be that sharply defined floral organ borders represent an ancestral state for all eudicots, but has been lost in some more derived lineages.

### Expression profiles of members of regulatory gene families

To gain further insights into the transcriptional activities of putative regulatory genes in floral development, we focused on gene families that are homologous to known regulators of plant development, particularly those encoding known or putative transcription factors. For convenience, we will refer to their predicted functions without using the words putative or predicted.

### MADS-box genes

Genes encoding proteins containing a MADS-box DNA binding domain represent the best-studied floral gene family, of which multiple members are crucial for floral development. In *E. californica *the expression of *EscaAG1 *(84248), *EscaAG2 *(86612), *EScaAGL2 *(87251), *EScaAGL9 *(87125), *EScaAGL11 *(89484), *EScaGLO *(87167), *EScaDEF1 *(83744) and *EScaDEF2 *(87005) have been studied with *in situ *hybridization [10,11,40,41]. Additionally, MADS-box genes homologous to those lacking characterized functions in *Arabidopsis *were included on our array, such as *EScaAGL54 *(87912). Expression of *EScaAGL54 *was highest in small buds, but showed similar levels in all the other tissues, suggesting a putative function in early floral stages.

To further understand the expression of the *E. californica *MADS-box genes, we plotted *E. californica *unigene expression profiles in comparison to the closest *Arabidopsis *homologs [11]. Expression patterns were largely similar between the two species, but there were some interesting differences (Figure 6). Both of the *E. californica AP3 *homologs showed similar expression patterns to *AP3*, differing only in that 87005 (*EscaDEF2*) showed lower expression in all tissues relative to 83744 (*EscaDEF1*) or *AP3 *in *Arabidopsis *(Figure 6a). At the same time, 87167 (*EscaGLO*), a homolog of *PI*, showed similar expression to *PI *in *Arabidopsis *(Figure 6b). Additionally, an *E. californica *homolog of the *Arabidopsis *C-function gene *AG *showed similar expression to that of *AG *(Figure 6c). Besides those key MADS-box genes regulating floral development, we found that *E. californica *homologs of E-function genes also have similar expression patterns to E-function genes in *Arabidopsis *(Figure 6d,e).

Homologs of other MADS-box genes demonstrated different expression patterns. Unigene 84248 (*EscaAG1*, an *AG *homolog [63]) was highly expressed in stamens and carpels as expected, while 86612 (*EscaAG2*, a second *AG *homolog [63]) exhibited similar levels of expression in all floral tissues, suggesting a divergent function for this gene in *E. californica *flower development (Figure 6c). Also, the homolog of *AGL6 *(86583) also showed higher expression in sepals and petals (Figure 6f), in contrast to the low expression of the *Arabidopsis AGL6 *gene in sepals on the basis of microarray expression [43]. Since a homolog of A-function gene has not been found in *E. californica*, it is possible that 86583 may function in the outer two whorls as an A-function gene (Figure 6f).

#### AGO

**Figure 7:**
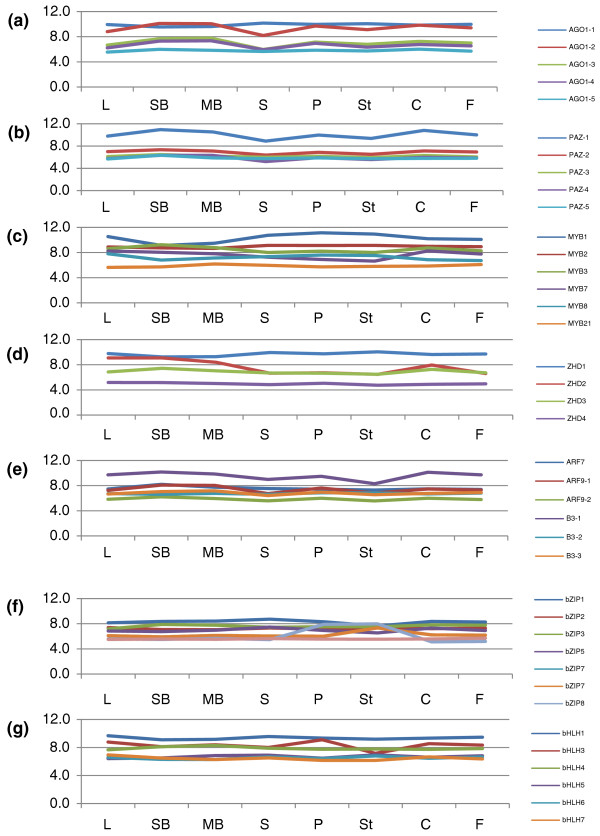
**The expression levels of members of the ARGONAUTE, MYB, Zinc-finger, Homeodomain, ARF, bZIP and bHLH families**. **(a)** The AGO gene family; **(b)** The PAZ gene family; **(c)** The MYB gene family; **(d)** The ZHD gene family; **(e)** The ARF gene family; **(f)** The bZIP gene family and **(g)** The bHLH gene family in eight tissues. All the expression values are log2 ratio. The same abbreviations of different tissues were used as in figure 5.

The ARGONAUTE (AGO) family is involved in RNA post-transcriptional regulation [64]. In *Arabidopsis*, members of the AGO family are involved in floral development, most likely through microRNA and small interfering RNA silencing. Our microarray included ten members of the AGO family, all of which were differentially expressed in at least one tissue (Figure 7a,b; expression data of family members are listed in Additional file 8). Among those genes, there was an interesting pattern, which identified three genes that were generally highly expressed in all organs while the remaining seven genes were expressed at moderate to low levels.

Among the genes examined in this study, three *AGO1 *homologs (one in the high expression group and two in the low expression group) shared similar expression patterns: twofold higher expression in petals, pre-meiotic and meiotic buds than in sepals. The *AGO *genes in *Arabidopsis *encode proteins with a PAZ domain (with nucleic acid binding activity [65]) and are expressed at similar levels in different tissues, except for PAZ-1, which was preferentially expressed in carpel, pre-meiotic and meiotic buds compared with sepal with more than twofold changes.

#### MYB

MYB transcription factors contain DNA binding domains and some have been identified as flower developmental regulators [66,67]. Eleven *E. californica MYB *genes were included on our microarray. Most *MYB *genes showed dramatic differential expression among tissues, but two were not differentially expressed among any of the tissues tested (Figure 7c; Additional file 8). One homolog of At4g32730 (*MYB1*) was expressed at higher levels in mature petals and stamens, suggesting that this gene may have a role in B function. A homolog of At4g32730 (*AtMYB3R1*) was significantly preferentially expressed (more than twofold higher) in the pre-meiotic bud in comparison with sepals, petals, and stamens and carpels. A homolog of At3g28470 (*AtMYB35*) was also preferentially expressed in pre-meiotic buds compared with all seven other tissues. A homolog of At4g01680 (*AtMYB55*) was significantly preferentially expressed in fruit in comparison with leaves, pre-meiotic and meiotic buds, petals, sepals and stamens. An At2g37630 (*AtMYB91*/*AS1*) homolog was more varied in expression but generally showed lower expression in stamens than in carpels, fruits, leaves, pre-meiotic and meiotic buds and lesser down-regulation in petals relative to carpels, leaves and pre-meiotic buds. Last but not least, a homolog of At3g61250 (*AtMYB17*) was expressed twofold significantly higher in meiotic buds compared with fruits.

#### Zinc Finger Homeodomain genes

Zinc Finger Homeodomain (ZHD) genes are expressed during floral development in *Arabidopsis *[68]. Our microarray contained four genes in this family. Two homologs of At1g75240 (ATHB33) were expressed without significant difference across all tissues. Of these two genes, one (88691) was expressed highly in both vegetative and reproductive organs while the other was barely expressed in all tissues, suggesting a functional divergence between these two paralogs (Figure 7d).

#### ARF

Auxin-response factors (ARFs) are believed to regulate auxin responsive genes [69,70]. This family contains *ETTIN *(At2G33860), a developmental regulatory gene that acts on regional identity in the perianth, stamens and carpels [71]. Most of the poppy *ARF *genes that were included on our microarray showed no differential expression among the tissues examined (Figure 7e). Only one gene, a homolog of At5g62000 (*ARF2*, 84471), showed twofold significantly different expression: twofold lower in stamens when compared with all tissues but sepal; and twofold lower in sepals compared with carpels, fruits and pre-meiotic buds.

#### bZIP

The bZIP (basic-leucine zipper) protein family contains the *Arabidopsis FD *(At4G35900, FD-1) and *PERIANTHIA *(At1G68640) genes, which are involved in flower development and the *HY5 *(At5G11260) gene involved in root development. Our array contained 12 members of this family, one of which was not differentially expressed among all tissues examined (Figure 7f; Additional file 8). From our microarray results, most of these genes showed only slightly different expression levels except the homologs of *bZIP7 *(83748) and *bZIP8 *(87035), both of which were expressed highly in stamens, with *bZIP8 *also highly expressed in petals. Previous studies of genes of the bZIP family suggested that some of them may act downstream of B-function genes to regulate floral development [72-74]. Because the homolog of *bZIP8 *was co-expressed with B-function genes, we speculate that this gene might have a function similar to that of the *Arabidopsis *homolog. In addition, a homolog of At4g38900 is expressed at a level twofold higher in sepals than in stamens.

#### bHLH

The basic helix-loop-helix family contains several *Arabidopsis *genes regulating flower development, including *SPATULA*, which controls the development of the carpel margins [75]. Eleven members of this family were included on our microarray, seven of which showed no significant differential expression (Figure 7g; Additional file 8). The other four genes demonstrated twofold differential expression among tissues examined. A homolog of At2g31210 (*bHLH91*, 89282) was most highly expressed in pre-meiotic buds and the expression level was at least twofold higher than in all the other tissues; also, its expression level in meiotic buds was at least twofold higher than any other floral organs. Since At2g31210 has an important role in anther development in *Arabidopsis *[45], its homolog in *E. californica *may function in a similar manner. Another gene, a homolog of At5g09460 (*bHLH143*), was also expressed at a higher level in the pre-meiotic buds than in sepals, petals and stamens and in meiotic buds. Additionally, this gene was expressed twofold higher in carpels and fruits than in stamens. A homolog of At1g26260 (*bHLH76*, *CIB5*) was expressed in pre-meiotic buds significantly twofold higher than in fruits and stamens. A homolog of At3g26744 (*bHLH116*/*ICE1*) was significantly down-regulated by twofold in stamens relative to carpels, fruits, leaves, meiotic buds and petals. This gene was also significantly more highly expressed by twofold in petals over sepals. The expression patterns of bHLH genes suggest that they might regulate several aspects of floral development and/or physiology, but are not necessarily associated with ABC functions. Further study of bHLH genes, and indeed many of the floral gene families examined here, in *Arabidopsis *and other species, including *E. californica*, may uncover their functions and reveal possible functional conservation among the eudicots.

## Conclusions

We examined transcriptome landscapes from eight tissues of the basal eudicot *E. californica *and identified preferentially expressed genes within and among floral developmental tissues, fruits and leaves. By comparing genes showing tissue-preferential expression patterns in *E. californica*, we found that genes preferentially expressed in specific reproductive organs or at certain stages tended to have less conserved expression levels compared with *Arabidopsis *than those preferentially expressed in leaves (Tables 1 and 2; Table S4 in Additional file 5). One possible explanation is that most of the leaf-preferential genes encode highly conserved chloroplast proteins.

We also identified the co-expressed and tissue-specific floral genes and characterized the signature of ABC domain genes. Our comparison of the gene expression patterns in *E. californica*, *Aquilegia*, *Persea *and *Arabidopsis *showed that the *E. californica *results support a 'sharp border' model, similar to that for core eudicots such as *Arabidopsis*, rather than the 'fading border' model in other basal angiosperms [29,62]. This is consistent with the clear morphological distinction of sepals and petals, and the lack of intermediate floral organs such as staminodes in *E. californica *flowers. In contrast, *Aquilegia *flowers have similar outer perianth organs and a distinct type of floral organ between stamens and the carpels, which is in good agreement with the microarray results of the floral organs [29]. Therefore, although both *E. californica *and *Aquilegia *are basal eudicots, the morphological and expression characteristics strongly suggested that they have divergent developmental programs, with *E. californica *more similar to core eudicots and *Aquilegia *resembling basal angiosperms. Our analysis of *E. californica *further suggested that flowers with distinct perianth organs might have originated at an earlier time than the ancestor of core eudicots. This study along with other works [21,29] highlight the importance of careful analysis of basal eudicots as an intermediate group of flowering plants to provide crucial information to bridge the gap between highly canalized core eudicots and morphological flexible basal angiosperms.

Our data also provide an overview of divergence and conservation between different species. The highly similar expression patterns of B- and C-function genes compared with the varied expression levels of other MADS-box genes in *Arabidopsis *and *E. californica *suggest that the conserved expression of only a few key genes may result in the high similarity of flower morphology between *Arabidopsis *and *E. californica. *The transcriptome analysis of other families with known functions in floral development indicates their possible roles in *E. californica*. Recent study of protein-protein interactions in basal eudicots (*Euptelea pleiospermum*, *Akebia trifoliata *and *Pachysandra terminalis*) suggested that MADS-box genes that interact with each other have co-evolved. This is most likely due to the fact that the majority of the protein-protein interactions are expected to be conserved to some extent to orchestrate floral architecture [76]. However, Zhao *et al*. [70] showed the AP1 lineage had a distinct interaction pattern; this, together with our results that *AGL6 *and *SEP *homologs are expressed in the A-domain, supports that A-function genes show less conservation [56]. In *Arabidopsis*, *AP1 *not only regulates the development of sepal and petal, but also integrates growth, patterning and hormonal pathways [77]. This dual function of *AP1 *observed in the core eudicots might be a more recent innovation that evolved since the divergence of the core from the basal eudicots.

Many of the genes showing tissue-specific expression noted in this study have homologs in *Arabidopsis *that are currently lacking in functional analyses. This study, when compared with similar studies in *Arabidopsis *and other species, should help us identify genes of interest that may play important, conserved roles in floral development [26,28,29]. We have identified a number of candidate genes that share similar expression patterns between *E. californica *and *Arabidopsis *but have not been functionally characterized. Our results suggest that *E. californica *has a similar floral program to the core eudicots, despite a mostly divergent set of genes outside of the MADS-box family. These results not only indicate that different regulatory machinery may operate among basal eudicots, but that canalized floral development might have originated prior to the core eudicots. Our findings also allow for informative comparisons with other species, allowing hypothesis formulation and stimulating further experimentation in model organisms, which now includes *E. californica*.

## Materials and methods

### Tissue collection and RNA isolation

Sixteen *E. californica *cv. 'Aurantica Orange' (JL Hudson Seedsman, La Honda, California, USA) plants were grown from seeds in a controlled greenhouse environment at the Pennsylvania State University (University Park, PA) under 16 hours light and watered and fertilized as needed. To avoid potential expression differences among collections due to circadian rhythms, leaves and floral tissues were only collected from individual plants between 8:30 and 10:30 am. Developing leaves of less than 5 mm length, developing fruits, pre-meiotic (small) buds less than 5 mm long, meiotic (medium) buds of 5 to 10 mm length and pre-anthesis sepals, petals, stamens and carpels were collected from 16 plants, immediately placed in liquid nitrogen and stored in a -80°C freezer until RNA extraction. Tissues from a group of four plants were then pooled to create one biological replicate, for a total of four replicates.

### Probe design for the *E. californica *transcriptome

To design oligonucleotide probes for *E. californica*, a two-stage pipeline for oligonucleotide probe design, Microarray Oligonucleotide Design and Integration Tool (MODIT) was used (probe information provided in Additional file 9). Briefly, MODIT integrates two existing programs: Array Oligo Selector (v.6; AOS) and OligoArray (v.8; OA), with subsequent independent evaluation and optimization steps. The pipeline enables one to design a set of probes having well-defined sequence and thermodynamic properties by first taking advantage of the strict thermodynamic criteria of OA to produce a partial set of optimized probes, and then filling in the set from among the large number of probes selected by AOS, after screening them for thermodynamic compatibility.

The MODIT pipeline screens candidate probes based on three parameters: high sequence specificity, appropriate melting temperature T_m_, and lack of stable secondary structure. The first criterion, sequence specificity, was determined using BLAST and Smith-Waterman local alignment tools to eliminate probes having a match to any non-target sequences of more than 15 consecutive nucleotides, or an overall match of more than 30 nucleotides [78-80]. The second criterion was that the probe set should have very similar T_m_. The MODIT user is informed of probes with T_m _outside a recommended range by flagging in the database, and she/he can decide whether to use such probes. A third criterion was the lack of stable secondary structure. MODIT allows values of probe ΔG_SS _above -0.5 kcal.mol^-1^, less than the energy of one hydrogen bond between bases [81]. We use melting temperature to independently recalculate a consistent set of thermodynamic properties for the probes and check for consistency [82]. The pipeline stores comprehensive information about probe thermodynamic properties and potential cross-reactions in a MySQL database, so that they can subsequently be used in array data analysis.

The MODIT pipeline was used to generate one 60-base probe for each gene in the 6,846 *E. californica *Unigene set [83,84], after masking regions that were conserved in multigene families in *Arabidopsis*, rice (*Oryza*) and *Populus*. Unigenes were sorted into gene families using PlantTribes [85] and conserved sites in the multiple sequence alignment were identified using the column score metric calculated by CLUSTAL [86]. A sodium concentration of 0.5 M was used in modeling of thermodynamic properties, following hybridization conditions recommended by Agilent for their 60-mer *Arabidopsis *Oligo Microarray Kit, and the conditions modeled by Lee *et al*. [87]. The probe concentration range that was used in the thermodynamics calculations is 2.44 mM following the calculations of Riccelli *et al*. [88] and assuming the default 1 nM target recommended in [89]. In the OA run, duplex melting temperature T_m _was constrained above 70°C, and the duplex T_m _for predicted cross-reactions and stable secondary structures was constrained below 60°C. For the AOS run, the constraint on GC content was maintained around 52%. Duplex melting temperature was constrained to keep 20°C separation between the upper and lower T_m _limits, to allow for selection of more candidate probes. The probe maximum and minimum match for non-target sequences were maintained at 15 and 10 nucleotides, respectively. When the two sets of probes were merged, the constraints applied to the merged set were: 80°C ≤ T_m _≤ 90°C, overall match with non-target as well as with consensus sequences should be less than 30 nucleotides and ΔG_SS _above -0.5 kcal.mol^-1^. Since one goal of this design was to obtain complete coverage of all target sequences, a selection of known suboptimal probes was added back to the final design (Additional file 9, column 5), and their sequence and thermodynamic properties tracked in the MODIT database. The design results obtained using MODIT for the target sequences from *E. californica *are summarized in Additional file 9. No application, including MODIT, could provide 100% target coverage while satisfying all of the design criteria for each probe. However, MODIT improved on target coverage and significantly limited potential cross-reactions relative to OA, while nearly eliminating probes that were predicted to form stable secondary structure.

Oligonucleotides of 60-bp length were designed from 6,446 *E. californica *unigenes obtained from a floral EST library [20] and cell culture suspension library [90]. Unigene builds were performed as described by Carlson *et al*. [20] and then sorted into putative gene families using the PlantTribes database [85]. Because the complete genome of *E. californica *is not yet sequenced, oligos were designed to specifically exclude conserved regions, when identified, so that expression analyses putatively represent single genes (see above). Oligonucleotide probes were arrayed on glass slides by Agilent (La Jolla, CA, USA).

### RNA extraction, microarray hybridization and scanning

RNA was isolated from eight tissues examined each with four biological replicate pools and cleaned using the RNeasy plant mini Kit (Qiagen, Valencia, California, USA) following Agilent's instructions. RNA concentrations were quantified using an Agilent 2100 Bioanalyzer and stored at -80°C before use, with yields of 20 to 35 micrograms of total RNAs from approximately 100 mg of tissues. Approximately 400 ng of total RNAs were used for cRNA synthesis with Cyanine 3-dCTP and Cyanine5-dCTP (Perkin-Elmer Life Sciences, Inc., Downers Grove, Illinois, USA) incorporation, using the Agilent Low RNA Input Kit (Agilent), according to the manufacturers' protocol. Qiagen's RNeasy mini-spin columns were used to purify amplified cRNA samples. Sample concentrations were quantified using a NanoDrop spectrometer (NanoDrop Technologies, Wilmington, Delaware, USA). Hybridization was performed using the In situ Hybridization Kit (Agilent) with 35 ng of Cy3- and Cy5-labeled cRNA following the manufacturer's instructions at 65°C for 17 hours. Prior to scanning, each slide was washed, rinsed and dried in Agilent's Stabilization and Drying Solution, as directed. Scanning was performed using a Gene Pix 4000A scanner and the Gene Pix Pro 3.0.6 Software (Axon Instruments (now Molecular Devices), Union City, California, USA) to produce two TIFF images at 532 nm and 635 nm. The microarray data have been submitted to the Gene Expression Omnibus database, with accession number [GEO:GSE24237].

### Statistical analyses of genes differentially expressed among tissues and developmental stages

Analyses were performed with the R programming language [91] and the limma package Bioconductor [92]. Arrays were background corrected and loess normalized within arrays and Aq normalized between arrays [93]. Agilent controls and other control probes were removed from the data. For the 93 *E. californica *oligos with multiple probes, we chose the probe with the highest 75% quantile value among the normalized 'A' intensities of all 16 arrays. A one-way single-channel empirical Bayes ANOVA was used to identify those genes [94,95] that were significantly differentially expressed among the seven floral RNAs and one leaf RNA examined, with an FDR of 0.05. Additionally, significant differences between combinations of more than one floral organ and leaf were also identified under the same parameters.

In order to identify those genes that were most likely to be organ/stage-specific in *E. californica*, we examined those genes with a significantly (FDR = 0.05) twofold greater expression in a single organ/stage relative to all other tissue stages examined. The expression of these genes was then compared to the expression, as determined by Affymetrix arrays [28], to their closest identified *Arabidopsis *homolog based on a tribe-MCL analysis, when available, to determine which genes may have conserved expression profiles. We were able to directly compare expression in pre-meiotic and meiotic buds in *E. californica *versus inflorescences containing stage 1 to 9 flowers in *Arabidopsis *(developing inflorescences), the *E. californica *fruit, capsules, versus the *Arabidopsis *fruit, siliques, *Arabidopsis *flowers at stage 12 nearing pre-anthesis versus sepals, petals stamens and carpels at anthesis in *E. californica *and genes preferentially expressed in leaves in both organisms.

### Real-time PCR experiments

To test the reliability of our microarray hybridizations, nine genes and one reference were investigated using quantitative RT-PCR. RNA (1 μg) of each tissue was treated with DNase (Invitrogen, Eugene, Oregon, USA), followed by reverse transcription using the Superscript III reverse transcriptase (Invitrogen). We then performed real time PCR using DyNAmo SYBR Green qPCR Kit from New England Biolabs (Ipswitch, Massachusetts, USA) under the following parameters: 95°C for 10 minutes, 40 cycles at 95°C for 30 s, 60°C for 1 minute. Fluorescence intensity was measured using Applied Biosystems' 7300 Sequence Detection System (Carlsbad, California, USA). Eca_2514 (Unigene84142) was chosen as the reference gene as it was not significantly differentially expressed among any of our examined tissues in the microarray experiments and it was expressed at a moderate level in all our tissues compared to all other genes. The relative amounts of cRNA converted from a messenger RNA was calculated using intensities corresponding to 'experimental' genes relative to the reference gene. We performed triplicate reactions for all tissues with samples containing no reverse transcriptase and no RNA as negative controls. All primer information is provided in Additional file 9.

## Abbreviations

AG: AGAMOUS; AGL: AGAMOUS-like gene; AGO: ARGONAUTE; AOS: Array Oligo Selector; AP: APETALA; ARF: Auxin-response factor; bHLH: basic helix-loop-helix; bZIP: basic-leucine zipper; DEF: DEFICIENS; ESca: *Eschscholzia californica*; EST: expressed sequence tag; FDR: false discovery rate; GLO: GLOBOSA; GO: Gene Ontology; MYB: Myeloblastosis-like gene; OA: OligoArray; PI: PISTILLATA; RT-PCR: real-time reverse-transcription PCR; TAIR: The *Arabidopsis *Information Resource.

## Authors' contributions

LMZ, HM, NSA, JLM and CWD designed the study; DT and LMZ performed tissue collection and RNA isolation; CJG and RG designed the oligonucleotides for the probes used in the microarray chip; XM, LMZ and DT performed RT-PCR experiments; LMZ, XM, NSA, QZ, and PKW performed data analysis; LMZ, XM, and HM wrote the manuscript drafts; LMZ, XM, HM, NSA, JLM, CWD, CJG, RG, and PKW edited the manuscript; all authors approved the manuscript.

## Supplementary Material

Additional file 1 **Supplemental figures**. Supplemental Figure 1: correlation coefficients between signal intensities from four biological replicates of seven tissues. Pearson's correlation coefficients were between 0.88 and 0.97 between any pair of the four biological replicates, indicating that the results were highly reproducible. Supplemental Figure 2: GO annotation pie chart of genes present across all tissues. GO categorization of all *Arabidopsis *homologs of poppy genes that were expressed across all the eight tissues with log2 values of signal intensity larger than 5.41 (10% percentile; control provided in Figure 4). Supplemental Figure 3: RT-PCR results consistent with microarray data. Nine genes were verified using RT-PCR. The lines in blue represent the RT-PCR results and red the microarray results. All the numbers shown in this figure are the fold changes of expression intensities in reproductive tissues compared with leaf. The left y-axis is for microarray results and right y-axis for RT-PCR results. Click here for file

Additional file 2 **Numbers of genes expressed in eight tissues using different cutoff and gene lists**. Table S1: a summary of numbers of genes expressed in eight tissues using different cutoff percentiles (5%, 10%, 15%). Table S2: genes expressed in both leaves and medium buds. Table S3: genes expressed in leaves, small buds and medium buds. Table S4: genes expressed in both leaves and small buds. Table S5: genes expressed in both small buds and medium buds. Table S6: genes expressed in leaves, sepals and petals. Table S6: genes expressed in both carpels and stamens. Table S7: genes expressed in either sepals and/or petals. Table S8: genes expressed in carpels and either sepals and/or petals. Table S9: genes expressed in carpels, stamens and either sepals and/or petals. Table S10: genes expressed in stamens and either sepals and/or petals. Click here for file

Additional file 3 **GO comparison between all genes on the chip and differentially expressed genes**. Gene numbers comparing all genes on the chip, genes expressed across different tissues and those differentially expressed between any two tissues in each GO category. Click here for file

Additional file 4 **Genes preferentially expressed in eight tissues and all the genes differentially expressed. **This additional file contains lists of all the genes preferentially and differentially expressed between any two tissues and lists of genes preferentially expressed in each tissue over all the other tissues. Column sequence, abbreviation and the version of annotation are the same as those used in Table 1 and all the other supplemental tables in Additional file 2. All the expression values are log2 ratio. Click here for file

Additional file 5 **Expression levels of *Arabidopsis *homologs of selected poppy genes**. This additional file contains information about the expression levels of *Arabidopsis *homologs of selected poppy genes of interest listed in the tables in our study. Click here for file

Additional file 6 **Genes identified as putative A-, B- and C-domain genes**. Click here for file

Additional file 7 **Genes preferentially expressed in reproductive tissues compared with leaf**. Click here for file

Additional file 8 **Gene expression of different gene families**. Click here for file

Additional file 9 **Probe design in the microarray and properties of *E. californica *probe sets designed by MODIT and other methods and primers used for RT-PCR experiments**. This additional file contains probe design and orientation of the custom microarray, properties of probe sets and primers for RT-PCR. Click here for file
