# Supplementary material for: Comparative transcriptomics among floral organs of the basal eudicot Eschscholzia californica as reference for floral evolutionary developmental studies
Source: Genome Biol. 2010 Oct 15;11(10):R101. doi: 10.1186/gb-2010-11-10-r101 (PMC3218657; doi:10.1186/gb-2010-11-10-r101)
Supplement: Additional file 1 — Supplemental figures. Supplemental Figure 1: correlation coefficients between signal intensities from four biological replicates of seven tissues. Pearson's correlation coefficients were between 0.88 and 0.97 between any pair of the four biological replicates, indicating that the results were highly reproducible. Supplemental Figure 2: GO annotation pie chart of genes present across all tissues. GO categorization of all Arabidopsis homologs of poppy genes that were expressed across all the eight tissues with log2 values of signal intensity larger than 5.41 (10% percentile; control provided in Figure 4). Supplemental Figure 3: RT-PCR results consistent with microarray data. Nine genes were verified using RT-PCR. The lines in blue represent the RT-PCR results and red the microarray results. All the numbers shown in this figure are the fold changes of expression intensities in reproductive tissues compared with leaf. The left y-axis is for microarray results and right y-axis for RT-PCR results. [file gb-2010-11-10-r101-S1.PPT]

## Slide 1
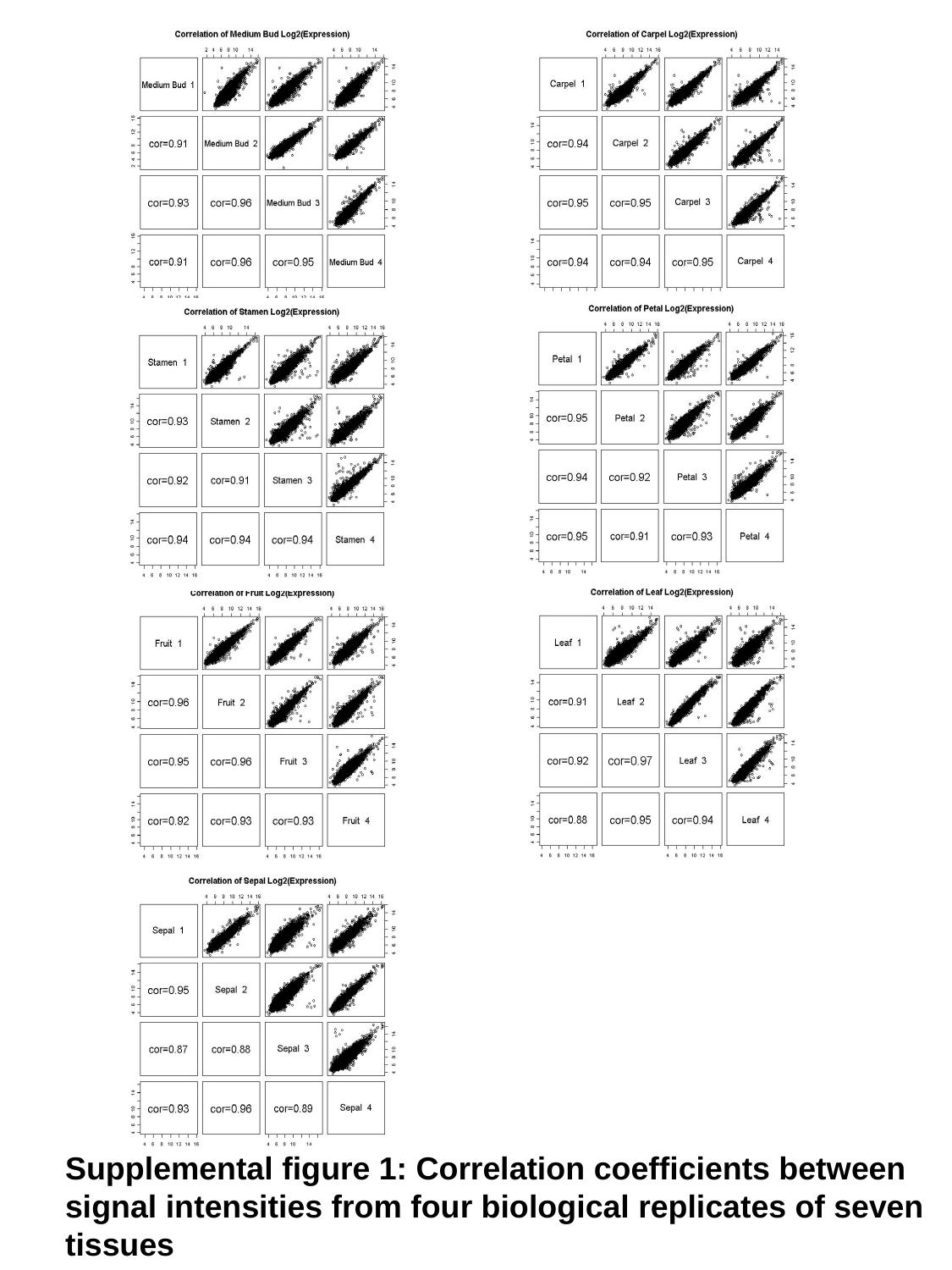

Supplemental figure 1: Correlation coefficients between signal intensities from four biological replicates of seven tissues

## Slide 2
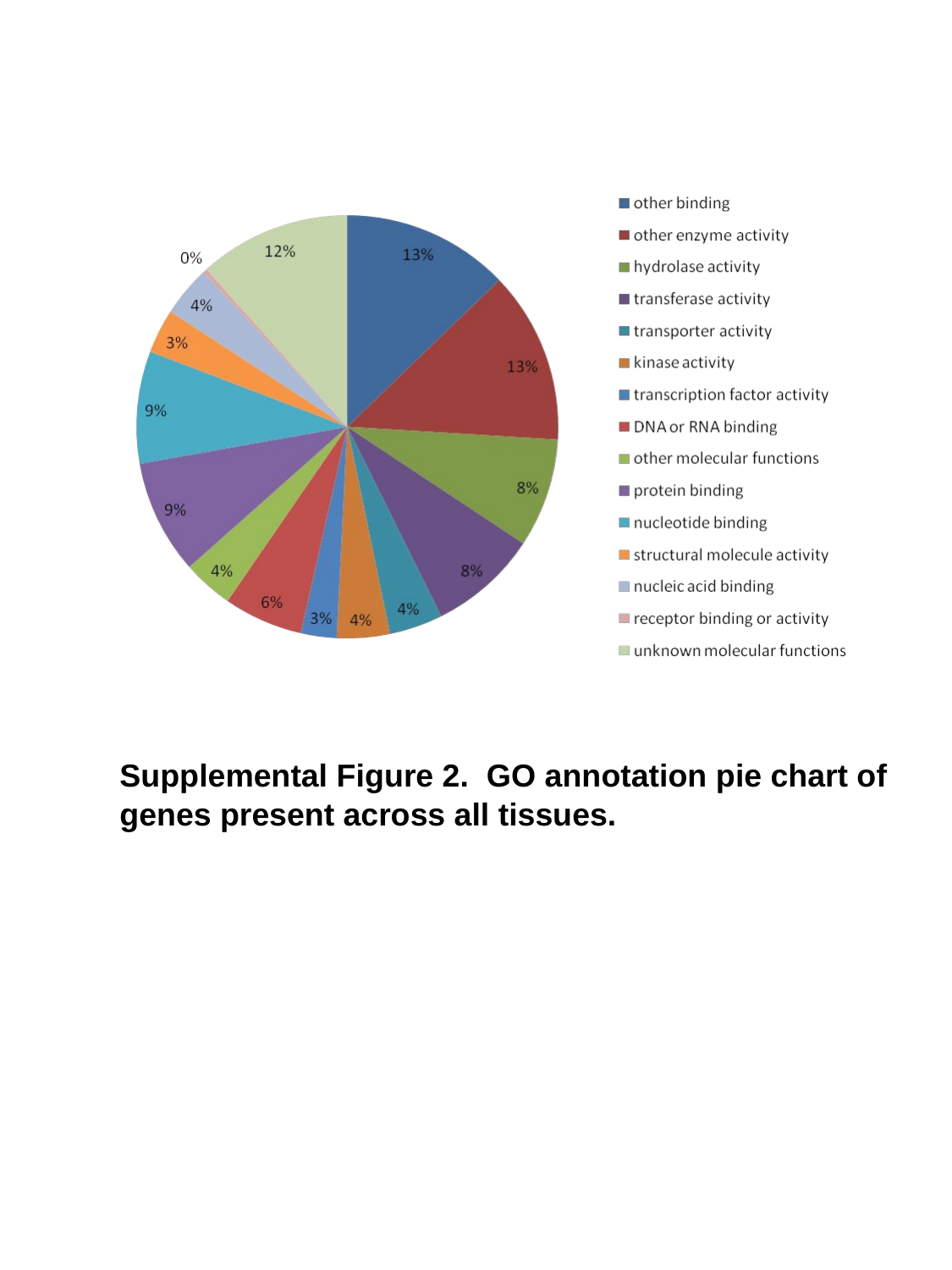

Supplemental Figure 2. GO annotation pie chart of genes present across all tissues.

## Slide 3
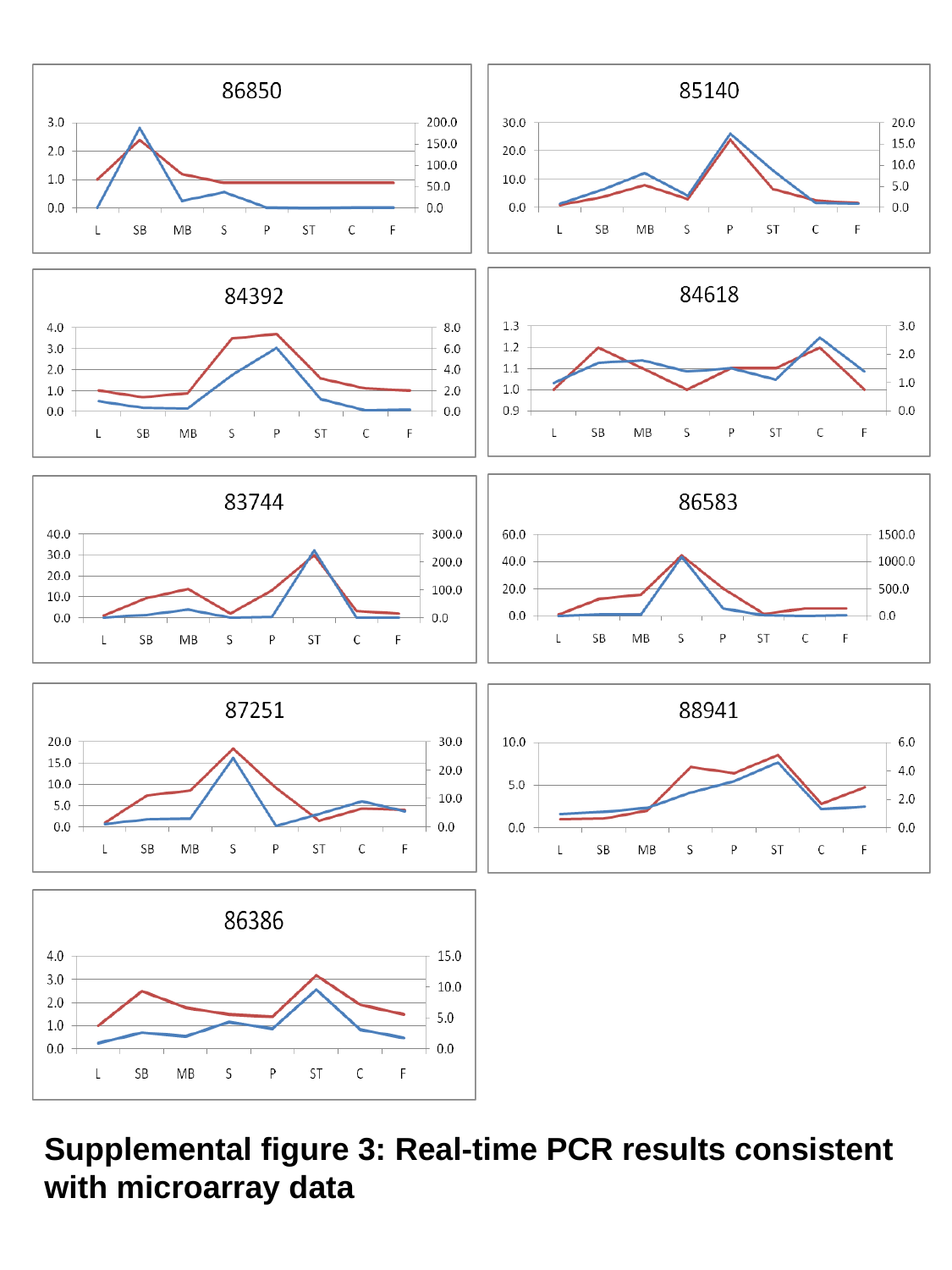

Supplemental figure 3: Real-time PCR results consistent with microarray data
